# Evaluation of a Tobacco Treatment Specialist Training Program Targeted to Behavioral Health Professionals

**DOI:** 10.3390/ijerph23050580

**Published:** 2026-04-30

**Authors:** Chizimuzo Okoli, Sarret Seng, Bassema Abu Farsakh, Emily Koyagi, Heather Beck, Audrey Darville

**Affiliations:** College of Nursing, University of Kentucky, Lexington, KY 40536, USA; sse255@uky.edu (S.S.); bassema.abufarsakh@uky.edu (B.A.F.); emily.koyagi@uky.edu (E.K.); herobe2@uky.edu (H.B.); audrey.darville@uky.edu (A.D.)

**Keywords:** tobacco treatment, behavioral health, workforce training, mental illness, tobacco treatment specialist, public health intervention

## Abstract

**Highlights:**

**Public health relevance—How does this work relate to a public health issue?**

- Tobacco use remains disproportionately high among adults living with mental illnesses, contributing to preventable morbidity and mortality, particularly in regions with high adult tobacco use prevalence rates.
- To address these tobacco-related disparities, Tobacco Treatment Specialist (TTS) training improves behavioral health providers’ competency to deliver evidence-based tobacco treatment.

**Public health significance—Why is this work of significance to public health?**

- A targeted, asynchronous TTS training program increased provider knowledge and confidence in delivering tobacco treatment within behavioral health settings
- The asynchronous TTS training initiative increased training accessibility for behavioral health providers, expanding the number of certified TTSs across participating community mental health centers from 0 to 55 over four years.

**Public health implications—What are the key implications or messages for practitioners, policy makers, and/or researchers in public health?**

- Flexible, web-based tobacco treatment training is a feasible strategy to strengthen behavioral health workforce capacity, though organizational support may be necessary to improve completion rates.
- Embedding trained TTSs within behavioral healthcare organizations may help reduce tobacco-related health disparities among people living with mental illnesses.

**Abstract:**

Tobacco use remains disproportionately high among adults living with mental illnesses (MIs), contributing to excess morbidity and mortality, particularly in high-burden states such as Kentucky. Behavioral healthcare providers are well-positioned to address this disparity but often lack formal training in evidence-based tobacco treatment. Tobacco Treatment Specialist (TTS) training provides healthcare providers with skills to deliver evidence-based tobacco treatment; however, traditional TTS training models often rely on synchronous, in-person or live virtual formats, which may limit accessibility for some healthcare workforces. Asynchronous, web-based delivery models may reduce these barriers. This study evaluates a targeted, asynchronous TTS training program designed for providers working in Community Mental Health Centers (CMHCs) across Kentucky between 2020 and 2024. Using a one-group post-test-only design to assess engagement and completion and a one-group pre-test–post-test design among completers, we examined program outcomes and changes in knowledge and attitudes. Eligible participants were nominated by the administrative team at each CMHC. Of 100 registered participants, 70.0% enrolled and 55.0% completed the program. Among completers (*n* = 55), knowledge scores increased significantly from pre- to post-test (t[51] = 7.6, *p* < 0.001). Participants also reported significant improvements in perceived skills (t[51] = 7.9, *p* < 0.001) and knowledge of resources to deliver tobacco treatment (t[51] = 7.8, *p* < 0.001), while perceived time to deliver services did not change significantly. These findings suggest that asynchronous TTS training is a feasible approach to improving tobacco treatment knowledge and confidence among behavioral health providers, though additional strategies may be needed to enhance completion and assess practice-level impact.

## 1. Introduction

For adults living with mental illnesses (MIs), tobacco use remains undertreated, and prevalence remains higher than that of the general adult population [1,2,3]. In the past several decades, although overall U.S. tobacco prevalence rates have substantially declined, there remains a gap in the prevalence between those with and without MIs. It is estimated that 33.4–65.0% of those with serious MIs use tobacco, a rate that is 2–3 times that of the general population (17.0%) [4,5]. This disparity is associated with high mortality rates from related cardiopulmonary disease and cancers in this population [6,7]. Moreover, tobacco use is associated with worsened mental health outcomes and often hinders MI recovery [8,9,10,11,12]. Hence, despite the overall gains from concerted tobacco control efforts in the U.S., tobacco use prevalence, related disease, and mortality remain disproportionately higher for those living with MIs and require targeted efforts to address the persistent gap.

Evidence-based tobacco treatment services are effective for supporting tobacco cessation among people with MIs. For example, recommended tobacco cessation pharmacotherapy (i.e., varenicline, bupropion, nicotine replacement products) is equally effective among those with or without MIs, but more intensive approaches are often required to optimize cessation in clinical practice [13,14]. Furthermore, various evidence-based psychotherapies, with or without the addition of tobacco cessation pharmacotherapy, are effective modalities of supporting cessation in these populations [15]. Despite clear recommendations for effective treatments, few behavioral healthcare providers either engage people with MIs in evidence-based tobacco treatment or are competent in the provision of tobacco treatment [16,17,18]. Low tobacco treatment engagement by and poor delivery of such evidence-based approaches to people with MI in behavioral healthcare settings continue to deprive this population from needed services. Thus, it is crucial to address this treatment gap with tobacco treatment training to support behavioral healthcare providers with the knowledge and confidence to deliver appropriate and effective tobacco treatment services to this population.

Despite the need for training approaches tailored to the clinical and contextual realities of behavioral healthcare, behavioral health providers remain underrepresented in traditional tobacco treatment training programs and often report limited access to training that is relevant to their practice settings. In particular, the Association for the Treatment of Tobacco Use and Dependence (ATTUD) endorses 11 core competencies for tobacco dependence treatment to strengthen healthcare provider training services [19]. Several studies have observed that tobacco treatment training programs can improve knowledge, attitudes, confidence, and skills in tobacco treatment delivery among healthcare providers [20,21,22]. However, a recent evaluation of accredited tobacco treatment training programs indicated a significant under-representation of behavioral health professionals trained in programs incorporating these core competencies, with only 12.0% working in addiction or counseling settings [23].

Furthermore, Tobacco Treatment Specialist (TTS) training programs are traditionally delivered through a combination of asynchronous pre-learning modules and synchronous, instructor-led sessions. While synchronous formats may support higher completion rates due to structured participation requirements, they can present barriers related to scheduling, staffing constraints, and competing clinical demands. Fully asynchronous models offer greater flexibility and accessibility, particularly for behavioral health providers working in resource-constrained settings; however, concerns remain regarding participant engagement and completion in the absence of structured, real-time interaction. Thus, given the disproportionate tobacco use burden among people with MIs, it is crucial to examine the outcomes of tailored evidence-based tobacco treatment approaches to improve knowledge and confidence in tobacco treatment delivery by behavioral healthcare professionals. The purpose of this study was to evaluate the outcomes of an innovative, virtual TTS training targeted to behavioral healthcare professionals. Among a sample of behavioral health providers, our specific aims were to evaluate:

1. Program engagement and completion.
2. Changes in knowledge and attitudes regarding tobacco treatment delivery among program completers.

## 2. Materials and Methods

### 2.1. Tobacco Treatment Specialist Program

Eligible participants completed the Council for Tobacco Treatment Training Program (CTTTP)-accredited BREATHE Online TTS course [20]. The BREATHE Online TTS course is based on ATTUD core competencies, which cover 11 key domains, including counseling, assessment, treatment planning, pharmacotherapy, ethics, relapse prevention, diversity and specific health issues, documentation and evaluation, professional resources, law and ethics, and professional development, which were structured in five modules [20]. The five modules cover counseling skills, treatment planning, pharmacotherapy, relapse prevention, and professional development and were tailored for relevance to behavioral health settings. Training modules were designed to be completed in a structured, sequential order; however, participants could revisit previously completed modules as needed. The CTTTP-accredited self-directed asynchronous TTS course is structured to be flexible, though on average, participants complete the training over six to ten weeks.

The training content was delivered through the Canvas© online learning platform and included a combination of didactic content, practice demonstration videos, written assignments with individualized feedback, quizzes, and supplemental resources tailored to participants’ needs. For example, supplemental resources were tailored to clinical roles and patient care for behavioral health practice settings, including materials relevant to specific patient populations, outpatient behavioral health settings, inpatient psychiatric care, and integrated care environments. To ensure competency, successful completion of the program requires a minimum of 27 contact hours completed through asynchronous training, with participants expected to pass module-specific quizzes, a comprehensive written case, a pharmacology exam, and a final exam with a minimum score of 75%. The program concludes with a synchronous simulated patient interaction with an instructor during a one-hour audio-video session, allowing participants to apply their acquired skills in a practical setting. Please see the methods described by Darville, Rademacher [20] for more in-depth details regarding the training program.

### 2.2. Procedures

Participants were recruited through professional networks, organizational outreach, and behavioral health agency partnerships. Recruitment was pragmatic rather than based on saturation, to engage a diverse range of behavioral health settings. We contacted the administrative leaders at 14 Community Mental Health Centers (CMHCs), as well as leadership at numerous other behavioral health organizations throughout the state, to determine interest in enrolling staff members in the behavioral TTS program. Program team members met with the administrative leaders at the inception of the targeted training and described the program, along with its requirements and its benefits to the organization. These initial meetings were followed up with email correspondence to provide further information about the program requirements and enrollment details.

The administrative team in each CMHC or behavioral healthcare organization identified and nominated eligible staff members to enroll in the program between December 2019 and April 2024. Participants were eligible if they were healthcare professionals, paraprofessionals, or staff working in behavioral health or related healthcare settings, including mental health, substance use treatment, or integrated care environments. Individuals not affiliated with healthcare or behavioral health service delivery were not eligible to participate. Then, the administrative staff at the CMHCs or behavioral healthcare organizations provided the names and contact information of their staff professionals who were recommended to participate in the program. Recommended participants were screened for eligibility, received detailed information about the requirements of the program, and provided demographic information. Participants who consented were then enrolled in the program. A total of 100 were enrolled in the program, representing 26 unique behavioral health organizations. The program was designed to be 6–8 weeks in duration, but each participant could self-pace. Participants who did not meet the completion timeline were contacted by email to determine any challenges and to encourage module completion. Participation required access to a computer or internet-enabled device; this access was not standardized across sites and may have been provided by either the participant or their organization. Organizational support varied by site and may have included encouragement from leadership; however, structured support (e.g., protected time or formal incentives) was not uniformly implemented. The training program was provided at no cost to participants.

### 2.3. Measures

Demographics: Information on participants’ age (in years), gender (male vs. female), and race/ethnicity (White vs. Black vs. other) was obtained. We assessed participants’ profession (medical [e.g., nurses, physician assistants, physicians] vs. non-medical [e.g., social workers, psychologists, peer support specialists, administrative staff]), and the practice setting (primarily mental health vs. primarily substance use/addiction treatment).

Program outcomes: Program outcomes were determined by examining engagement and completion, and changes in knowledge and attitudes related to tobacco treatment.

Program engagement and completion: All enrolled participants were required to complete a questionnaire on their knowledge and attitudes regarding tobacco use and treatment before and after program completion. The training platform did not include proctoring or monitoring features to verify independent completion of quizzes, and responses were therefore based on participant self-directed engagement. Program engagement was defined by participants who enrolled in the course and completed the pre-test questionnaire before commencing the program. Completion was defined as additionally completing all required program components and a post-test questionnaire at the end of the program. We also examined the duration of time to complete the program (in weeks).

Changes in knowledge and attitudes: We determined changes in knowledge related to tobacco use and treatment using a 20-item questionnaire consisting of true/false statements [20]. Examples of knowledge questions included: “Tobacco is the deadliest addictive substance”, “Most people start smoking as young adults”, and “Standardized cessation treatments work better than tailored treatments”. Scores ranged from 0 to 20, with higher scores indicating a greater degree of knowledge. In addition, attitudinal questions consisted of three items using a five-point Likert scale from “0” being Strongly Disagree to “4” being “Strongly Agree”. Participants were asked to indicate their level of agreement with the statement, “I have the skills needed to deliver effective tobacco treatment services”, “I am knowledgeable of the resources needed to deliver effective tobacco treatment services”, and “I have the time to deliver effective tobacco treatment services”.

### 2.4. Data Analysis

Descriptive statistics using means (*M*) and standard deviations (*SD*) or frequencies (*n*) with percentages (%), as appropriate, were employed to examine the participants’ demographic information. Program engagement and completion were determined by using means (*Ms*) with standard deviations (*SDs*) or frequencies (*n*) with percentages (%). Demographic differences between those who completed or did not were determined using independent sample *t*-tests for continuous variables and chi-square analyses for categorical variables. We further described the program engagement and completion rates by cohort using frequencies and percentages. Furthermore, among completers, paired sample *t*-tests were used to assess changes in knowledge and attitude scores before and after the training program. All analyses were conducted using IBM-SPSS version 29 with an alpha-level of 0.05 for significance.

## 3. Results

### 3.1. Sample Characteristics

Of 100 individuals who registered for the program, 30 did not enroll, while 70 enrolled and initiated training. Most were 36 years of age or older (62.0%), female (76.0%), and white (82.0%). Nearly half held a master’s degree or higher (49.5%). Participants represented an interdisciplinary behavioral health workforce, including both medical (e.g., nurses, nurse practitioners, advanced practice providers, respiratory therapists, pharmacists) and non-medical roles (e.g., mental health counselors, substance use counselors, social workers, health educators, and public health professionals), all of whom were engaged in behavioral health or related care settings. Most worked in non-medical job roles (66.0%), in mental health clinics (37.0%), or combined mental health and substance use settings (20.0%). Moreover, the majority worked in urban locations (74.0%). Of these, 70.0% of participants were engaged, and 55.0% completed the program.

Among those who completed the program (*n* = 55), the majority were 36 years of age or older (60%), female (82.6%), and white (89.1%). More than half held a master’s degree or higher (54.4%). Most worked in non-medical job roles (72.7%) in a mental health clinic (45.5%). Additionally, the majority worked in an urban location (81.8%). On average, those completing the program did so in a median of 15.15 weeks (ranging from 5.30 to 74.30 weeks, *SD* = 16.7). Detailed data on partial module completion are unavailable due to limitations in data tracking. See Table 1.

### 3.2. Participant Characteristics and Completion Status

There were no significant associations between age group (χ^2^[2] = 0.91, *p* = 0.63), gender (χ^2^[2] = 1.16, *p* = 0.46), work location (χ^2^[2] = 3.94, *p* = 0.14), ethnicity (χ^2^[2] = 2.70, *p* = 0.26), medical versus non-medical discipline (χ^2^[2] = 2.91, *p* = 0.23), and type of facility (χ^2^[6] = 7.85, *p* = 0.25) with completion status. There was a significant association between cohort start date and completion status (χ^2^[8] = 32.78, *p* < 0.001, Cramer’s V = 0.41), indicating meaningful differences in completion rates across program cohorts. There was also a significant association between education level and completion status (χ^2^[6] = 18.45, *p* = 0.005, Cramer’s V = 0.30). Participants with lower educational attainment (associate’s degree or less) were less likely to complete the program, whereas those with bachelor’s and graduate degrees had higher completion rates.

### 3.3. Changes in Knowledge Scores

Among participants who completed the program (*n* = 55), there was a significant increase in knowledge scores, by an average of 3.4 points (*SD* = 3.2), at post-test (*M* = 17.0, *SD* = 2.6) compared to pre-test (*M* = 13.8, *SD* = 2.0, t[51] = 7.6, *p* < 0.001, Cohen’s d = 1.1)

### 3.4. Changes in Attitude Scores

A series of paired samples *t*-tests was conducted to assess changes in self-perceived skills, knowledge of resources, and time to deliver effective tobacco treatment services from pre- to post-course completion. Participants reported significantly greater perceived skills after the training (pre-test *M* = 2.2, *SD* = 1.1; post-test *M* = 3.5, *SD* = 0.5), a mean increase of 1.31 points (t[51] = 7.9, *p* < 0.001, Cohen’s d = 1.1). Similarly, perceived knowledge significantly improved from pre-test to post-test (pre-test *M* = 1.9, *SD* = 1.1; post-test *M* = 3.4, *SD* = 0.9), with a mean increase of 1.40 points (t[51] = 7.8, *p* < 0.001, Cohen’s d = 1.08). However, there was no significant difference in perceptions of having sufficient time to deliver treatment services between pre-test (*M* = 2.8, *SD* = 0.9) and post-test (*M* = 2.9, *SD* = 1.0, t[51] = 0.7, *p* = 0.47).

## 4. Discussion

The present study evaluated outcomes among healthcare providers who participated in an asynchronous, web-based TTS training program across behavioral healthcare organizations in Kentucky. Among the behavioral health organizations targeted for recruitment, the program enrolled 100 participants with a 55.0% program completion rate. Among those who completed the program, we observed significant improvements in tobacco use and treatment knowledge, as well as in self-perceived skills and confidence to deliver tobacco treatment services. Together, these findings indicate the feasibility of a tobacco treatment training targeting behavioral healthcare providers.

Completion rates observed in the present study are lower than those reported in several highly structured tobacco treatment training programs but may be comparable when considering differences in delivery modality, participant composition, and institutional support [21,24]. For example, the MD Anderson Tobacco Treatment Training Program reported a 91% completion rate among 1155 healthcare professionals trained between 2017 and 2023; like our program, completion for this program was defined by successfully passing a formal examination within a credentialing framework for TTSs [21]. That program primarily enrolled frontline medical providers, most commonly nurses and nurse practitioners, whose clinical roles explicitly allow more opportunities for tobacco assessment and treatment, and who may therefore experience stronger professional incentives to complete training. Likewise, the parent BREATHE Online TTS course enrolled 210 participants from a variety of settings between 2017 and 2019, of which 92% completed the training [20]. Although both the parent BREATHE TTS program and the program evaluated in the current study share asynchronous delivery and credentialing requirements, differences in participant composition (i.e., general healthcare providers vs. mental and behavioral healthcare providers), organizational context, and implementation expectations may account for variability in completion rates.

In addition to individual program evaluations, workforce-level factors may further contextualize these findings. An examination of CTTTP-accredited TTS training programs reported that 7761 trainees completed TTS training programs between 2016 and 2019 [23]. While completion rates cannot be calculated because of a lack of enrollment information, most CTTTP-accredited programs during this period relied on in-person workshop-based delivery in medical or academic settings. These environments may foster higher completion through fixed schedules, peer accountability, and institutional endorsement. Therefore, it is important to consider that while asynchronous delivery improves accessibility, it may also reduce accountability and peer engagement, typically reinforced in in-person workshop-based delivery in synchronous learning environments. Additionally, variability in digital access and technological resources may have influenced participants’ ability to complete the training, although these factors were not directly measured. Furthermore, a critical distinction between the present study and several prior training initiatives is the degree of institutional support for post-training implementation. For example, approximately 20% of trainees in the CTTTP-accredited TTS training program reported being sent by their employers, and many pursued TTS certification with explicit intentions to implement tobacco treatment services or obtain certification [23], suggesting the crucial role of organizational support and professional motivation in sustaining engagement. Additionally, the Canadian Training Enhancement in Applied Counselling and Health (TEACH) Project enrolled 741 interprofessional providers and required supervisory or organizational commitment to implement tobacco treatment practices as a condition of participation. Although framed differently from traditional completion metrics, 55.0% of participants were implementing tobacco treatment, and 91.0% were engaged in knowledge transfer activities at six-month follow-up, further suggesting that organizational endorsement may serve as a significant motivator for program completion and sustained engagement [22]. This structural support likely contributed to program completion as a salient motivator. In our cohort, the absence of such organizationally required commitment may have reduced perceived role relevance and institutional reinforcement for completion, potentially contributing to lower completion rates. Although organizational leadership supported recruitment and participation, this form of support may differ from ongoing structural support (e.g., organizational commitment to implement tobacco treatment practices, protected time, or workload adjustments) that facilitates program completion. The absence of sustained organizational reinforcement may contribute to lower completion rates in asynchronous training environments.

Another distinction that may have influenced the outcomes of our program was workforce composition. Most participants in our cohort represented a range of behavioral health professionals, including mental health counselors, social workers, health educators, and substance dependence counselors. While these roles are essential to tobacco treatment delivery within behavioral health settings, professionals who do not have prescribing authority or medication management roles may perceive fewer opportunities, structural support, or professional incentives to complete training. In contrast, tobacco treatment training programs that predominantly enrolled nurses, nurse practitioners, physicians, and other medical staff (whose clinical responsibilities routinely include tobacco screening and treatment) may demonstrate higher completion rates due to stronger role alignment, institutional expectations, and direct integration of tobacco treatment into routine care. For example, the MD Anderson Tobacco Treatment Training Program primarily trained nurses and nurse practitioners and reported high completion rates [21]. Similarly, the Canadian TEACH program enrolled largely nurse-based interprofessional teams [22]. Furthermore, the CTTTP-accredited TTS training programs report indicated that most trainees are embedded in medical or academic settings [23], where institutional expectations and role alignment may support training engagement and completion. It is also important to note that these comparison programs were delivered online using synchronous formats, whereas the present program was fully asynchronous. While delivery modality may contribute to differences in completion, these findings further support that workforce composition and alignment with clinical roles remain key factors influencing engagement in tobacco treatment training.

A unique aspect of the tobacco training program used in our study was the web-based format with an asynchronous training schedule, the first of its kind [20]. This delivery method yields participation and completion rates that are like other training modalities for behavioral health providers, with some studies report higher enrollment in virtual training than in in-person training [24]. Additionally, asynchronous delivery offers several advantages for healthcare workers, including flexibility to engage with content around clinical schedules, reduced need for protected time, and the ability to progress at an individualized pace. In the present study, this approach yielded moderate enrollment and completion rates, as well as significant improvements in tobacco treatment knowledge and attitudes, suggesting that asynchronous training can be both feasible and effective for mental and behavioral health providers. These findings can be contrasted with other virtual tobacco treatment training programs that rely on synchronous delivery formats. For example, Rutgers University offers a virtual, synchronous, two-week tobacco treatment training program targeted to behavioral health providers working in substance use and addiction settings [25]. This program utilizes real-time instruction, group discussion, and structured scheduling, which may enhance accountability and engagement through live interaction with instructors and peers. However, completion rates and longitudinal engagement outcomes for this program have not been published, limiting direct comparison with asynchronous models. Nevertheless, the existence of this program highlights an alternative virtual approach that prioritizes synchronous participation over flexibility.

It is important to note that these training cohorts were conducted before the widespread availability of generative artificial intelligence (AI) tools; however, the increasing accessibility of AI introduces new considerations for assessment integrity in asynchronous learning environments. Future training programs may consider incorporating strategies such as scenario-based assessments, time-limited quizzes, or reflective application exercises to better evaluate participant understanding. Additionally, incorporating platform-level monitoring tools or adaptive assessment designs may help mitigate potential threats to independent knowledge assessment in fully asynchronous training models.

We also found improvements in knowledge and attitudes towards tobacco treatment and delivery, including increased confidence in the skills and knowledge necessary to deliver tobacco treatment. Similar findings have been observed in several studies evaluating tobacco treatment training programs [20,21,22]. These improvements did not differ by the type of healthcare provider or the treatment setting. In other words, delivering tobacco treatment training to different healthcare providers in behavioral health settings may not require any further tailoring to provider type or setting. This finding further suggests that such programs can be used with the goal of improving knowledge and attitudes towards tobacco treatment engagement. Notably, the operationalization of “attitudes” in this study combined perceived knowledge, confidence, and available time, which may differ from traditional conceptualizations of attitudes in the literature. As such, comparisons with other studies should be interpreted with caution.

A few important limitations must be acknowledged to aid in interpreting the findings of this study. First, as the selection of participants was purposive, we cannot easily generalize our findings to populations of healthcare providers in behavioral healthcare contexts beyond our settings. The sample was predominantly female, White, and master’s educated, which may reflect patterns in workforce composition and leadership selection for professional development opportunities. These characteristics may not fully reflect the demographic diversity of individuals who use tobacco, particularly among populations disproportionately affected by tobacco use. The targeted enrollment was a minimum of 28 participants for cohorts 1–3 and 14 participants for cohorts 4–5 over the five-year implementation period, with reduced cohort sizes in later years due to financial limitations. Although we cannot provide an exact estimate of the percentage of the mental and behavioral health programs in Kentucky represented by the participating organizations, the TTS participants represent all 14 state-funded community mental health center regions of Kentucky. Future studies employing random selection of participants may provide more reliable information on the generalizability of these findings. Second, the advent of the COVID-19 pandemic introduced additional stressors related to workload and staff shortages, which may have impacted some program engagement and completion rates among certain cohorts enrolled in the program. Examples of challenges were that some program participants changed jobs or had competing organizational priorities (i.e., staffing shortages, COVID-19-oriented prevention projects/interventions). Third, improved knowledge and attitudes as found in our evaluation do not necessarily translate to tobacco treatment delivery or actual engagement of individuals with MIs. Data linking methods (e.g., chart audits, surveys of program participants after program completion) in future studies may strengthen the evaluation of the impact of such provider training on individuals with MIs and within behavioral health settings. Finally, the non-medical category included a range of roles; however, administrative and non-clinical positions were not separately identified, limiting the ability to assess whether engagement or completion differed by role type. Future research should differentiate clinical and administrative roles to better understand engagement patterns in asynchronous training.

## 5. Conclusions

Despite the inherent limitations of the evaluation study, some strengths include the changes in knowledge and attitudes as intended outcomes. In four years, the number of certified TTSs in participating CMHCs increased from 0 to 55. Future research, employing qualitative and quantitative methods, is needed to assess the impact of the training program on the delivery of tobacco treatment within participating behavioral healthcare organizations. Such studies will be important in guiding strategies to address the disproportionately high tobacco-related morbidity and mortality and poor treatment engagement observed among people with MI. These findings underscore the need for continued investment in behavioral health workforce capacity building to address tobacco use among populations with disproportionately high burdens.

## Figures and Tables

**Table 1:** Chi-Square Tests of Independence for Completion Status by Participant Characteristics.

Variable	Total	Completed (*n*, % Within Completion Status)	Engaged Pretest Only (*n*, % Within Completion Status)	Non-Engaged (*n*, % Within Completion Status)
Program Engagement Status	100	55	15	30
Age				
≤35 years	34 (35.4%)	21 (38.9%)	3 (25.0%)	10 (33.3%)
36 years or older	62 (64.6%)	33 (61.1%)	9 (75.0%)	20 (66.7%)
Missing	4			
Gender				
Male	16 (17.4%)	7 (13.2%)	3 (25.0%)	6 (22.2%)
Female	76 (82.6%)	46 (86.8%)	9 (75.0%)	21 (77.8%)
Missing	8			
Work Location				
Rural	26 (26.0%)	10 (18.2%)	5 (33.3%)	11 (36.7%)
Urban	74 (74.0%)	45 (81.8%)	10 (66.7%)	19 (63.3%)
Ethnicity				
White	82 (83.7%)	49 (89.1)	10 (76.9%)	23 (76.7%)
Other	16 (16.3%)	6 (10.9%)	3 (23.1%)	7 (23.3%)
Missing	2			
Job Role				
Medical	22 (25%)	9 (18.4%)	4 (40.0%)	9 (31.0%)
Non-Medical	66 (75%)	40 (81.6%)	6 (60%)	20 (69.0%)
Missing	12			
Type of Work Facility				
Mental Health Clinic	37 (37.0%)	25 (45.5%)	5 (33.3%)	7 (23.3%)
Substance Abuse Facility	11 (11.0%)	6 (10.9%)	0 (0.0%)	5 (16.7%)
Both Mental Health and Substance Abuse	20 (20.0%)	9 (16.4%)	5 (33.3%)	6 (20.0%)
Other	32 (32.0%)	15 (27.3%)	5 (33.3%)	12 (40.0%)
Cohort Start Date *				
12 February 2020	30 (30.0%)	19 (34.5%)	8 (53.3%)	3 (10.0%)
28 April 2021	24 (24.0%)	12 (21.8%)	0 (0.0%)	12 (40.0%)
27 April 2022	11 (11.0%)	4 (7.3%)	0 (0.0%)	7 (23.3%)
26 April 2023	17 (17.0%)	13 (23.6%)	0 (0.0%)	4 (13.3%)
10 April 2024	18 (18.0%)	7 (12.7%)	7 (46.7%)	4 (13.3%)
Educational Attainment **				
Associate’s degree or less	15 (15.0%)	3 (5.5%)	2 (13.3%)	10 (33.3%)
Bachelor’s degree	30 (30.0%)	20 (36.4%)	4 (26.7%)	6 (20.0%)
Master’s or Graduate degree	49 (49.0%)	30 (54.5%)	6 (40.0%)	13 (43.3%)
Prefer not to answer/Missing	6 (6.0%)	2 (3.6%)	3 (20.0%)	1 (3.3%)

* *p* < 0.001, ** *p* < 0.01.

## Data Availability

Data will be made available upon reasonable request by the primary author.

## Abbreviations

The following abbreviations are used in this manuscript:

MIs	Mental Illnesses
ATTUD	Association for the Treatment of Tobacco Use and Dependence
CTTTP	Council for Tobacco Treatment Training Programs
BREATHE	Bridging Research Efforts and Advocacy Toward Healthy Environments (a research, outreach, and practice collaborative of the University of Kentucky College of Nursing)
TTS	Tobacco Treatment Specialist
CMHCs	Community Mental Health Centers
TEACH	Training Enhancement in Applied Counselling and Health
